# Editorial: Online Adaptive MR-Guided Radiotherapy

**DOI:** 10.3389/fonc.2021.748685

**Published:** 2021-08-30

**Authors:** Linda G. W. Kerkmeijer, Vincenzo Valentini, Clifton D. (Dave) Fuller, Ben J. Slotman

**Affiliations:** ^1^Department of Radiation Oncology, Radboudumc, Nijmegen, Netherlands; ^2^Department of Radiation Oncology, Catholic University of the Sacred Heart, Rome, Italy; ^3^Department of Radiation Oncology, MD Anderson Cancer Center, Houston, TX, United States; ^4^Department of Radiation Oncology, Amsterdam UMC, Amsterdam, Netherlands

**Keywords:** MRI, external beam radiotherapy, MR-guided radiotherapy, adaptive, image-guided radiotherapy

The radiotherapy field is rapidly evolving due to advances in radiation delivery and image guidance. After the introduction of image guided radiotherapy (IGRT) two decades ago, the integration of magnetic resonance imaging (MRI) with linear accelerators is the next logic step in IGRT. MR-guided radiotherapy will lead to a paradigm shift in radiation oncology for multiple clinical indications in the head and neck, thorax, abdomen and pelvis and opens new opportunities to increase precision and to adapt the treatment (1–3).

In this Research Topic, the opportunities and challenges when using online adaptive MR-guided radiotherapy will be described. Online adaptive magnetic resonance guided radiotherapy (MRgRT) has the potential to improve both oncologic outcomes due to dose escalation and ultra-hypofractionation, and decrease toxicity due to improved targeting accuracy by inter- and intrafraction adaptation. However, the adaptive workflow is also time and resource intensive and requires a drastic transformation of the offline and online radiotherapy workflow (4, 5).

Two MRI linear accelerator (MR-linac) systems to deliver MRgRT are commercially available and have been clinically implemented across the world, other systems are being developed. The technical specifications, opportunities and challenges of these MRgRT platforms (Elekta Unity and Viewray MRIdian) are described by Thorwarth and Low. In order to reduce time and resources per treatment fraction, automatization of most of the realtime MRgRT workflow is necessary. Before routine implementation of these technical solutions, large standardized data sets including both clinical and technical data are required for training and clinical validation of these models.

Although differences across both platforms are present and for few indications one system may have advantages over the other, in general, both systems offer new functionality, including MR-guidance and online adaptation when compared to conventional CT-guided radiotherapy. With increasing implementation of MRgRT systems, the time window for high quality comparative (randomized) trials is narrow, as described by Verkooijen and Henke. International collaborative studies, preferably across platforms, are warranted to gain this timely evidence of the superiority of MRgRT including patient-reported endpoints. For both systems, international research consortia have been formed, where expert clinicians, physicists, methodologists, therapists and technologists join forces for an evidence-based introduction of the technology and optimize the clinical impact (6). Large international prospective data registries collecting clinical and technical data are being set-up aiming to include all patients treated at the MR-linac for an evidence-based introduction of the technology and further evolution of the technology (7).

When introducing complex innovations in radiation oncology, a randomized controlled trial will not be the first step after clinical implementation of the novel technology. Several preparatory steps are required before comparative studies can be initiated, especially with a continuously evolving technology and evolving clinical application. The R-IDEAL framework describes the steps towards an evidence-based introduction of the new technology, with Phase 0 (Radiotherapy predicate studies), Phase 1 (Idea, first in man study), Phase 2a (technical development studies), Phase 2b (Exploration, early effectiveness in randomized studies), Phase 3 (Assessment in comparative studies) followed by Phase 4 (Long-term results) (8). Furthermore early Health Technology Assessments of resource-intensive treatments would help facilitate the reimbursement policy.

Besides the online adaptive approach and MR-guidance during treatment, one of the unique aspects of MRgRT, is the opportunity to perform biology-based image guided adaptive radiotherapy (BIGART) as described by Van Houdt et al. By acquiring biological images revealing metabolic and functional data, focal dose escalation to the gross tumor volume within the clinical target volume can be pursued, for example for prostate cancer (9). Also this opens opportunities for ‘dose painting by numbers’ within the tumor volume by using the heterogenic characteristics within the tumor to deposit a differentiated dose per voxel (10). MRgRT allows for daily quantitative imaging by visualizing the tumor volume, shape and biology before and during each radiotherapy fraction. Adaptation to changing tumor shape and volume is already possible in present-day MRgRT. In addition, imaging biomarkers need to be identified that can predict treatment response early in the course of treatment, which may eventually lead to response-adapted radiotherapy. Again, large multicenter (standardized) imaging and clinical data with multicenter and multiple tumor site validation are necessary, which further strengthens the importance of collaboration in large international data registries.

While BIGART may potentially impact all radiotherapy (+/- systemic therapy) applications, it may be of particular importance for the enhancement of radio-immunotherapy. The immunogenic effect of radiation and its synergy with immunotherapy, has been observed in several pre-clinical and clinical studies and in pre-clinical studies the dose per fraction seemed to be critical (11). As MRgRT allows for safe ultra-hypofractionation and reduces the volume of normal tissue irradiated by reduced treatment margins, radio-immunotherapy delivered by MR-guidance may be a perfect match. Furthermore, MRgRT facilitates visualization of the anatomical sites that should or should not receive radiation, allowing for new clinical treatment paradigms such as partial tumor irradiation or draining lymph node sparing. Many questions need to be addressed such as radiation dose, fractionation, timing of radiotherapy versus immunotherapy, target volumes and biomarkers for response prediction as highlighted by Hörner-Rieber et al. It should not be a surprise, that international collaboration, standardization and clinical and imaging data collection, including biomaterial, will be the driving force towards optimization of this combination treatment and proving its impact on oncological outcomes.

Since 2015, MR-linacs have been first used and were implemented across the world from initial users to now dozens of early adopters (12, 13). After the predicate and first in man studies, for several clinical indications studies have been performed on the early effectiveness, toxicity and patient reported outcomes of MRgRT. In this editorial, several review articles on MRgRT to treat tumors in the brain and spine, head and neck, lung, esophagus, pancreas, kidney, liver, cervix, prostate, bladder and rectum) are presented, including an overview of the current evidence, clinical experience, state of the art implementation of MR-guided radiotherapy and future perspectives (Boldrini et al.; Boeke et al.; Tocco et al.; Crockett et al.; Boldrini et al.; Maziero et al.; Lee et al.; Hall et al.; Keller et al.; Hijab et al.; Portelance et al.).

Although clinicians see the great potential of MRgRT as a logical next step in IGRT, and the first studies support the potential benefit of MRgRT, randomized clinical evidence is not yet available. A collaborative international effort (across platforms) to set up comparative trials or prospective registry studies will be necessary in the generation of high quality evidence on the benefits of MRgRT over CT-guided radiotherapy.

With this Research Topic on online adaptive MR-guided radiotherapy, we aim to give the reader an overview of the ongoing advances in MR-guided radiotherapy to facilitate institutes on the verge of implementation of MR-guided radiotherapy into clinical practice. We thank all authors for their excellent invited reviews and their willingness to collaborate across platforms and share their expertise on MR-guided radiotherapy. We believe that MR-guided radiotherapy can have a tremendous impact on outcomes for patients for multiple oncological indications. Advances in image-guided adaptive and response-based radiotherapy are expected to translate into improved oncologic outcomes, increasing the number of indications to be treated by stereotactic radiotherapy as a non-invasive treatment modality, reducing toxicity and reducing the impact of cancer treatment on quality of life. Therefore, we would like to make a ‘warm plea’ for international and across platforms collaboration of experts involved in the multidisciplinary teams of MR-guided radiotherapy to maximize the benefit of this paradigm shift in radiation oncology and to prove its superior outcome and cost-utility for the radiotherapeutic treatment of cancer patients rather sooner than later.

## Author Contributions

All authors were involved in the conception and design of this editorial. LK drafted the manuscript. All authors contributed to the article and approved the submitted version.

## Conflict of Interest

**LK:** The Radboudumc and UMC Utrecht are members of the MR-linac Consortium. Elekta financially supports the MR-linac Consortium and its member institutes in their research programs. **VV:** declares participation in a company sponsored speaker’s bureau and receipt of grants/research supports: AMGEN, ASTELLAS, ASTRAZENECA, BAYER, BRISTOL MYERS SQUIBB, EISAI, FERRING, IPSEN, ISTITUTOGENTILI, JANSSEN-CILAG, MSD, MERCK, NORGINE, NOVARTIS FARMA, PFIZER, ROCHE, SANOFI, SERVIER ITALIA, VARIAN, ELEKTA, VIEWRAY. **DF:** has received federal funding and salary support unrelated to this project from the, including: the National Institutes of Health (NIH) National Institute for Dental and Craniofacial Research Establishing Outcome Measures Award (1R01DE025248/R56DE025248) and an Academic Industrial Partnership Grant (R01DE028290); NCI Early Phase Clinical Trials in Imaging and Image-Guided Interventions Program (1R01CA218148); an NIH/NCI Cancer Center Support Grant (CCSG) Pilot Research Program Award from the UT MD Anderson CCSG Radiation Oncology and Cancer Imaging Program (P30CA016672) and an NIH/NCI Head and Neck Specialized Programs of Research Excellence (SPORE) Developmental Research Program Award (P50 CA097007); National Science Foundation (NSF), Division of Mathematical Sciences, Joint NIH/NSF Initiative on Quantitative Approaches to Biomedical Big Data (QuBBD) Grant (NSF 1557679); NSF Division of Civil, Mechanical, and Manufacturing Innovation (CMMI) standard grant (NSF 1933369) a National Institute of Biomedical Imaging and Bioengineering (NIBIB) Research Education Programs for Residents and Clinical Fellows Grant (R25EB025787-01); the NIH Big Data to Knowledge (BD2K) Program of the National Cancer Institute (NCI) Early Stage Development of Technologies in Biomedical Computing, Informatics, and Big Data Science Award (1R01CA214825). Direct infrastructure support is provided to Dr. Fuller by the philanthropic gift of the Stiefel Oropharyngeal Research Fund of the University of Texas MD Anderson Cancer Center Charles and Daneen Stiefel Center for Head and Neck Cancer, the Cancer Center Support Grant Radiation Oncology/Cancer Imaging Program and Multi-Investigator Research Program (both *via* P30CA016672), and the MD Anderson Program in Image-guided Cancer Therapy. DF has received direct industry grant support, honoraria, and travel funding from Elekta AB. **BS:** AmsterdamUMC has research grants for Varian medicial systems and ViewRay. I have received honorarium from ViewRay.

## Publisher’s Note

All claims expressed in this article are solely those of the authors and do not necessarily represent those of their affiliated organizations, or those of the publisher, the editors and the reviewers. Any product that may be evaluated in this article, or claim that may be made by its manufacturer, is not guaranteed or endorsed by the publisher.
